# Systemic and local immunosuppression in glioblastoma and its prognostic significance

**DOI:** 10.3389/fimmu.2024.1326753

**Published:** 2024-02-28

**Authors:** Aleksei A. Stepanenko, Anastasiia O. Sosnovtseva, Marat P. Valikhov, Anastasia A. Chernysheva, Olga V. Abramova, Konstantin A. Pavlov, Vladimir P. Chekhonin

**Affiliations:** ^1^ Department of Fundamental and Applied Neurobiology, V. P. Serbsky National Medical Research Center of Psychiatry and Narcology, the Ministry of Health of the Russian Federation, Moscow, Russia; ^2^ Department of Medical Nanobiotechnology, Institute of Translational Medicine, N. I. Pirogov Russian National Research Medical University, The Ministry of Health of the Russian Federation, Moscow, Russia; ^3^ Center for Precision Genome Editing and Genetic Technologies for Biomedicine, Engelhardt Institute of Molecular Biology, Russian Academy of Sciences, Moscow, Russia

**Keywords:** glioblastoma, glioma, lymphopenia, macrophages, myeloid-derived suppressor cells, natural killer cells, neutrophil-to-lymphocyte ratio, regulatory T cells

## Abstract

The effectiveness of tumor therapy, especially immunotherapy and oncolytic virotherapy, critically depends on the activity of the host immune cells. However, various local and systemic mechanisms of immunosuppression operate in cancer patients. Tumor-associated immunosuppression involves deregulation of many components of immunity, including a decrease in the number of T lymphocytes (lymphopenia), an increase in the levels or ratios of circulating and tumor-infiltrating immunosuppressive subsets [e.g., macrophages, microglia, myeloid-derived suppressor cells (MDSCs), and regulatory T cells (Tregs)], as well as defective functions of subsets of antigen-presenting, helper and effector immune cell due to altered expression of various soluble and membrane proteins (receptors, costimulatory molecules, and cytokines). In this review, we specifically focus on data from patients with glioblastoma/glioma before standard chemoradiotherapy. We discuss glioblastoma-related immunosuppression at baseline and the prognostic significance of different subsets of circulating and tumor-infiltrating immune cells (lymphocytes, CD4+ and CD8+ T cells, Tregs, natural killer (NK) cells, neutrophils, macrophages, MDSCs, and dendritic cells), including neutrophil-to-lymphocyte ratio (NLR), focus on the immune landscape and prognostic significance of isocitrate dehydrogenase (*IDH*)-mutant gliomas, proneural, classical and mesenchymal molecular subtypes, and highlight the features of immune surveillance in the brain. All attempts to identify a reliable prognostic immune marker in glioblastoma tissue have led to contradictory results, which can be explained, among other things, by the unprecedented level of spatial heterogeneity of the immune infiltrate and the significant phenotypic diversity and (dys)functional states of immune subpopulations. High NLR is one of the most repeatedly confirmed independent prognostic factors for shorter overall survival in patients with glioblastoma and carcinoma, and its combination with other markers of the immune response or systemic inflammation significantly improves the accuracy of prediction; however, more prospective studies are needed to confirm the prognostic/predictive power of NLR. We call for the inclusion of dynamic assessment of NLR and other blood inflammatory markers (e.g., absolute/total lymphocyte count, platelet-to-lymphocyte ratio, lymphocyte-to-monocyte ratio, systemic immune-inflammation index, and systemic immune response index) in all neuro-oncology studies for rigorous evaluation and comparison of their individual and combinatorial prognostic/predictive significance and relative superiority.

## 1 Introduction

In the United States, glioblastoma (diagnosed during 2013–2017) accounts for 48.6% (all ages combined) of all malignant brain and other central nervous system tumors, and five-year survival for patients diagnosed with glioblastoma in 2009–2015 is 7% (3–27%, varied by age) (1). Although the incidence of glioblastoma among primary brain tumors in different countries varies greatly (from ≈8.5% to 69%) (2, 3), five-year relative survival estimates are comparable and are <7% overall (4). The standard of care for the treatment of glioblastoma (maximal safe resection followed by radiotherapy with temozolomide chemotherapy) has not changed much since 2005 (Stupp protocol) (5, 6), and glioblastoma remains incurable (1) despite significant advances in our knowledge of its genetics and molecular biology over the past two decades.

The blood brain barrier is thought to be a key factor limiting the effectiveness of chemotherapy, including targeted agents, in the treatment of glioblastoma/glioma (7–9). Even temozolomide, with features such as 100% oral bioavailability, rapid absorption, excellent biodistribution, and ability to cross the blood-brain barrier because of its small size and lipophilic properties (10), reaches levels in tumor tissue that are only 20% of systemic drug levels (11). In 2016, of the ongoing 98 phase I/II and II glioma clinical trials, 63 studies (64.29%) were reported to include at least one drug able to pass the blood brain barrier (12). Unfortunately, according to systematic reviews and meta-analyses, almost all clinical trials involving targeted drugs and personalized chemotherapy in adult patients with glioblastoma have been unsuccessful (13–17). However, recent phase II trials (NCT02684058 and NCT04775485) found that dabrafenib plus trametinib could be an effective therapy as first-line treatment for pediatric patients with low-grade glioma with *BRAF* V600 mutations (18), and type II RAF inhibitor tovorafenib could be an effective therapy for BRAF-altered, relapsed/refractory pediatric low-grade glioma (19). Moreover, patients with *IDH1* wild-type high-grade gliomas harboring *BRAF* or *NF1* mutations and receiving trametinib monotherapy or in combination with dabrafenib had longer progression-free and overall survival than patients who did not receive genotype-matched targeted therapy (20).

Great hopes are currently placed on combination immunotherapy, including oncolytic virotherapy (16, 21–25). The immune system plays a primary role in the control of tumor development and the effectiveness of anticancer therapy (26–28). Historically, the brain has been considered an immune-privileged organ based on the lack of traditional lymphatic vessels in the brain and experiments with transplantation of foreign tissue into brain tissue and lack of rejection, as well as experiments with peripherally injected dyes that stain peripheral organs, but not the brain, due to the blood-brain barrier, restricting the access of macromolecules and cells into the brain parenchyma (29). Increasing evidence demonstrates that meningeal lymphatic vessels draining to the cervical lymph nodes play an important role in immune surveillance of the brain (30–32) and are essential for mounting an efficient immune response to brain tumors (33, 34). Various types of immune cells, including T cells and dendritic cells, were observed within meningeal lymphatics in both normal and pathological conditions (30–32). In mice with intracranial glioma or metastatic melanoma, dorsal meningeal lymphatic vessels were found to undergo extensive remodeling, and their specific pharmacochemical ablation impaired intratumoral fluid drainage, dendritic cell trafficking, and the efficacy of immunotherapy (33). Despite the partial disruption of the blood-brain barrier in glioblastoma (7), which promotes the infiltration of immune cells (25), nevertheless, human glioblastoma exhibits a predominantly “cold” (“immune-desert”/”immune-excluded”) phenotype, characterized by the absence or exclusion of T cells in the tumor microenvironment (33, 35) and T cell dysfunction, including tolerance and exhaustion (36, 37).

A tumor subdues the immune system, exerting both a local complex inhibitory effect on the tumor tissue microenvironment and systemic immunosuppression through the secretion of many soluble factors (38, 39). Profound immunosuppression and lymphopenia pose a challenge to current treatment strategies, including chemotherapy (38, 40, 41) and especially immunotherapy, the effectiveness of which may critically depend on the state of the patient’s immune system (41, 42). This review focuses on understanding the state of the immune system and the prognostic significance of different immune cell subtypes in patients with glioblastoma before standard therapy. In our accompanying review in *Frontiers in Immunology* (43), we comprehensively discuss the prognostic significance of standard therapy-related (iatrogenic) systemic immunosuppression and its implications for immunotherapy and oncolytic virotherapy. We provide compelling clinical data indicating that standard therapy affects various immune cell subsets, promoting tumor-related immune deficiency in patients with glioblastoma. Low post-treatment total lymphocyte count (TLC) is a prognostic factor for shorter survival in glioblastoma, and radiation-induced lymphopenia is a prognostic factor for mortality in virtually all solid cancers. Chemotherapy and corticosteroids may exacerbate radiation-induced lymphopenia. Dexamethasone use is a prognostic factor for shorter survival in glioblastoma. In addition, there is growing evidence that immunosuppression associated with standard therapy may be a barrier to immunotherapy, and lymphopenia is significantly associated with response and survival outcomes in patients with advanced cancer receiving immune checkpoint inhibitor therapy. Finally, we discuss how detailed blood and/or tumor immunophenotyping may be valuable for immunotherapy/oncolytic virotherapy research in terms of identifying new or validating the proposed immunological-based prognostic/predictive variables, and suggest what changes/interventions to the standard therapy paradigm should be considered to maintain lymphocytes counts. These reviews should help inform more rational clinical trial design and treatment decisions to potentially improve the effectiveness of immunotherapy/oncolytic virotherapy.

## 2 Glioma/glioblastoma-related changes in circulating immune cells

Analysis of peripheral blood obtained from patients with glioma showed shifts in the normal CD4+/CD8+ T cell ratio (from 2:1 closer to 1:1) (44–46). Among 300 chemoradiotherapy- and surgery/biopsy-naïve patients with glioblastoma (median age: 66; range: 21–91), lymphopenia (<1000 cells/µl) was present in 24.7% of patients (18.2% of steroid-naïve and 37.1% of steroid-experienced) (47). Deng et al. reported that 11.9% (out of n=469) of patients with glioblastoma (median age: 60.3; range: 19–94) had grade 3/4 lymphopenia (<500 cells/µl) preoperatively and 15.4% (out of n=628) postoperatively and before standard radiochemotherapy (48). In the elderly group (median age: 71 years), only 57% (out of n=72) of patients had normal baseline total lymphocyte counts (49). In another study, lymphopenia at baseline was detected in 24.3% (out of n=562) of elderly patients with glioblastoma (≥65 years) and was associated with worse overall survival (HR 1.30; 95% CI 1.05–1.62; *p*=0.02), regardless of O-6-methylguanine-DNA methyltransferase (*MGMT)* promoter methylation status (50). Similarly, studies reported that lymphopenia or absolute lymphocyte count at baseline in patients with glioblastoma were associated with worse overall survival in univariate and multivariate analysis, independent of the extent of resection, *IDH* mutation status, and adjuvant therapy (51, 52). However, absolute lymphocyte count at baseline was not correlated with overall survival in univariate or multivariate analysis of other studies (53–59).

In addition to baseline (preoperative/pretreatment) lymphopenia and shifts in the CD4+/CD8+ T cell ratio, it has been repeatedly documented that patients with glioma have decreased serum levels of Th1-type cytokines (IL-2, IL-6, IL-12, TNF-α, and IFN-γ) and increased serum levels of Th2-type cytokines (IL-4 and IL-10) (46, 60–64). Serum, cerebral spinal fluid, or tumor cyst fluid from patients with glioma may suppress the proliferation and/or function of lymphocytes and other immune cells from healthy donors (61, 63, 65, 66).

One of the contributors to systemic immunosuppression is CD4+CD25+FOXP3+ regulatory T cells (Tregs), which are involved in immune tolerance of tumors and compromise cytotoxic T cell function (67). In the majority of studies, elevated Treg fractions were documented among peripheral blood CD4+ T cells of patients with glioblastoma (60, 68–70), even in cases with severe CD4+ T cell lymphopenia (<200 cells/μL) and regardless of steroid use (70). Patients with elevated Treg fractions but not normal Treg fractions showed significant proliferative dysfunction of CD4+ T cells, reduced quantities of Th1-type cytokines, and increased quantities of Th2-type cytokines (70).

Another contributor to systemic immunosuppression is myeloid-derived suppressor cells (MDSCs), a heterogeneous population of early myeloid progenitors and precursor cells that can suppress immune responses mediated by CD4+ and CD8+ T cells (71). The number of circulating MDSCs is higher in patients with glioblastoma than in healthy donors or patients with low-grade gliomas (72–75).

In contrast to changes in the counts of T cell subsets, MDSCs, and neutrophils (discussed below), natural killer (NK) cells (45, 46, 76–78) and natural killer T (NKT) cells (79, 80) were within the normal range in patients with glioblastoma/glioma before standard therapy in the majority of studies, although their cytotoxic activity may have been impaired (44, 81, 82).

## 3 A high neutrophil-to-lymphocyte ratio is an independent prognostic factor for shorter survival

### 3.1 Factors influencing the ratio of neutrophils and lymphocytes

Neutrophils account for 50–70% of circulating leukocytes in humans (83). The neutrophil-to-lymphocyte ratio (NLR), derived from the absolute neutrophil and lymphocyte counts of a full blood count, is an easily accessible and measurable marker (84). Changes in the balance between neutrophils and lymphocytes reflect an increase in systemic inflammation and a decrease in anti-tumor adaptive immunity (84). Baseline NLR increases with glioma progression (grade I-IV glioma), with the highest NLR values observed in patients with grade IV gliomas, followed by grade III and grade I-II gliomas (85–92). In addition, in a retrospective study of adult patients with not otherwise specified subtype of glioblastoma (n=89), a weak positive association was found between tumor size and preoperative NLR values (Spearman r=0.3212, p=0.0493) (93). In another retrospective study of patients with glioma (n=64), higher pretreatment NLR was significantly associated with larger tumor diameter (p=0.02) (94). Similarly, a positive correlation between NLR and tumor size in patients with papillary thyroid carcinoma has been repeatedly documented (95). In carcinomas in general, NLR is higher in patients with more advanced or aggressive disease, as evidenced by increased tumor stage, nodal stage, and number of metastatic lesions (84). This correlation might be due to the fact that tumor cells secrete granulocyte colony-stimulating factor (G-CSF) and/or granulocyte-monocyte colony-stimulating factor (GM-CSF), which are not only direct growth factors for tumor cells but may also contribute to increased NLR in patients, shifting bone marrow hematopoiesis from the lymphocyte lineage toward the granulocyte lineage (96–98).

Any type of damage to brain tissue, including surgery- and therapy-related damage, tends to enhance G-CSF/GM-CSF synthesis (96). In addition, patients with glioblastoma treated with steroids have higher neutrophil counts and NLR (48, 54, 55, 78, 99, 100); however, no significant influence of steroid use on neutrophil counts (101) or only a weak correlation between dexamethasone dose and NLR (53, 57) were also reported. It is advisable to measure NLR prior to surgery or other treatments that may increase the neutrophil count. It should also be noted that NLR may change not only under the direct influence of tumor progression, surgery, or steroid treatment, but also of local or systemic infection; inflammatory diseases; thyroid, renal, or hepatic dysfunction; diabetes mellitus; heart diseases; hypertension; obesity; psychologic stress; and other complications in cancer patients (102, 103).

Regional anesthesia for patients with glioma has been proposed as a strategy to reduce postoperative systemic and local inflammatory responses (104). In a retrospective study of patients with glioblastoma (n=119) (104), local anesthesia of the nerves of the scalp during craniotomy, referred to as a “scalp block” (105), was shown to reduce postoperative NLR (104). This reduction was associated with longer median progression-free survival (16.7 *versus* 6.5 months for patients without a scalp block) (104). However, in another retrospective large cohort study (n=808), the use of a scalp block in glioma resection was not associated with improved progression-free and overall survival (106). Moreover, the use of different anesthetics, including isoflurane, desflurane, and propofol, during glioblastoma surgery is not associated with overall survival (107, 108).

### 3.2 A high neutrophil-to-lymphocyte ratio is a prognostic factor in solid tumors

In retrospective studies, lower neutrophil counts before radiochemotherapy were associated with better overall survival (n=164, n=369, and n=2002) (55, 57, 99) independent of steroid use (55). Higher neutrophil counts at relapse were also prognostic for worse overall survival, but only in patients who did not receive bevacizumab (109). However, there are studies that report no prognostic role for neutrophil counts in patients with glioblastoma (52, 56, 58, 59).

Higher NLR was a significant prognostic factor for shorter progression-free survival in some glioblastoma/glioma retrospective studies (110–113) but not in others (51, 54, 114–116) on univariate and/or multivariate analyses. In large meta-analyses, higher NLR in patients with carcinomas was a significant prognostic factor of cancer-specific, progression-free and/or disease-free survival (117, 118).

High baseline (preoperative/pretreatment) NLR was established as an independent predictor of shorter overall survival in patients with glioblastoma/gliomas (**Table 1**) (51, 52, 54, 56–59, 85, 90–92, 112, 114, 119–128), and this was confirmed by meta-analyses (131–133). However, the independent prognostic significance of NLR remains debatable (55). High NLR during standard therapy was also associated with worse overall survival regardless of steroid use in multivariate analysis (48), and a decline of NLR during or post-therapy was associated with longer overall survival of patients with glioblastoma in multivariate analyses in prospective (53) and retrospective studies (57, 119, 134). NLR was prognostic in patients with recurrent glioblastoma (119, 135). However, in some glioblastoma studies, baseline NLR was not correlated with overall survival (114, 136), or correlated with overall survival in univariate analysis but not in multivariate analysis (55, 56, 85, 127), or *vice versa* (121). Similarly, postoperative NLR was not correlated with overall survival in either univariate (56, 58) or multivariate analyses (57, 115, 127). It should also be noted that assessment of dynamic changes of NLR (e.g., preoperative, pre-treatment, during and/or post-treatment) may provide more accurate prognostication or prediction of response to therapy in glioma (53, 57) and carcinoma (137–144).

In the majority of glioblastoma/glioma studies (**Table 1**), high NLR was established as an independent factor for worse outcomes without including *MGMT* promoter methylation status, steroid use, *IDH* mutation status, or other prognostic variables in multivariate analyses. Nevertheless, baseline NLR was still prognostically independent of *MGMT* promoter methylation status (58, 129), *IDH* mutation status (51, 92, 121), and steroid use (122, 124) in patients with glioblastoma, and patients with increased NLR and requiring steroids had the poorest outcomes (73). Moreover, in a recent prospective glioma study (n=73, 37% with grade III astrocytoma and 63% with grade IV glioma), patients were divided into four groups based on the median baseline NLR and NLR decrease during chemoradiotherapy (53). Patients with baseline NLR <3.5 with NLR decrease during treatment (n=14), baseline NLR <3.5 without NLR decrease during treatment (n=23), baseline NLR ≥3.5 with NLR decrease during treatment (n=24), and baseline NLR ≥3.5 without NLR decrease during treatment (n=12) had median overall survival of 36.5, 19.2, 14.7 and 7.1 months, respectively (53). On univariate analysis, patients with baseline NLR <3.5 and NLR decrease during treatment had lower mortality risk than those with baseline NLR ≥3.5 (HR 0.512; 95% CI 0.291–0.904) or no decrease during treatment (HR 0.519; 95% CI 0.293–0.918) (53). Moreover, NLR decrease during treatment was a significant predictor of overall survival on multivariate analysis [HR 0.380; 95% CI 0.18–0.80)] after adjustment for age, ECOG performance status, extent of resection, *IDH* mutation status, grade IV tumor, and baseline and time-weighted mean dexamethasone dose (53).

**Table 1:** High NLR is an independent prognostic factor for overall survival in patients with glioblastoma/gliomas.

Study (year)	Patients	A NLR value associated with overall survival	Correlation with overall survival in univariate and multivariate analysis	Limitations/comments
Bambury et al. (2013) (59)	• Total: 84 • Male: 65 (77%); female: 19 (23%) • Median age: 58 (18-79) • Grade IV: 84 (100%)	• >4 vs. ≤4 • Median NLR (range): 3.1 (1.1-34.6) • NLR >4: 30 (35.7%); NLR ≤4: 54 (64.3%)	• 7.5 vs. 11.2 months, HR 1.6, 95% CI 1.00-2.52, p=0.048 • Multivariate: HR 1.81, 95% CI 1.08-3.01, p=0.025	• Retrospective • Relatively small sample size • No data on steroid use, *MGMT* promoter methylation status, and *IDH* mutation status (patients diagnosed between 2004 and 2009) • Neutrophil and lymphocyte counts in isolation were not prognostic • Multivariate analysis adjusted for age, gender, ECOG performance status, extent of resection, tumor location, full Stupp protocol, and second line therapy
Alexiou et al. (2014) (112)	• Total: 51 • Male: 30 (58.8%); female: 21 (41.2%) • Mean age: 59.2 ± 14.2 • Grade IV: 51 (100%)	• >4.73 vs. ≤4.73 • Mean NLR: 6.7 ± 4.6 • NLR >4.7: 29 (56.8%); NLR <4.7: 22 (43.2%)	• 11 vs. 18.7 months, p=0.01 • Multivariate: 95% CI 1.4-17.3, p=0.011	• Prospective • Relatively small sample size • No data on steroid use, *MGMT* promoter methylation status, and *IDH* mutation status (patients diagnosed between 2007 and 2013) • No data on confounding variables in multivariate analysis
McNamara et al. (2014) (119)	• Total: 107 (95 analyzed) • Male: 76 (71%); female: 31 (29%) • Median age (range): 52 (20-76) • Grade IV: 100%	• >4 vs. ≤4 • Median NLR (range): 6 (1.3-27.7) • NLR >4: 60 (63.2%); NLR ≤4: 35 (36.8%)	• 5.9 vs. 9.7 months (p=0.02); TR 1.86, 95% CI 1.18-2.93, p=0.01 • Multivariate: TR 1.65, 95% CI 1.15-2.35, p<0.01	• Retrospective • Relatively small sample size • Blood sampling time: post-therapy prior to second surgery • 67.3% of patients used steroids prior to second surgery • No data on *MGMT* promoter methylation status and *IDH* mutation status (patients diagnosed between 2004 and 2011) • No data on confounding variables in multivariate analysis
Han et al. (2015) (58)	• Total: 152 • Male: 95 (62.5%); female: 57 (37.5%) • Mean age: 50.4 ± 15.4 • Grade IV: 152 (100%)	• ≥4 vs. <4 • Mean NLR: 4.1 ± 3.8; Median NLR (range): 2.54 (0.7-20.6)	• 10.6 ± 9.8 vs. 17.9 ± 11.0 months, HR 2.139, 95% CI 1.464-3.125, p<0.001 • Multivariate: HR 2.068, 95% CI 1.304-3.277, p=0.002	• Retrospective • No data on *IDH* mutation status (patients diagnosed between 2010 and 2014) and steroid use • NLR ≥4 was associated with increased tumor neutrophil infiltration/decreased CD3+ infiltration • Neutrophil and lymphocyte counts in isolation were not correlated with survival • Multivariate analysis adjusted for age, KPS, extent of resection, and *MGMT* promoter methylation status
Auezova et al. (2016) (85)	• Total: 178 • Male: 93 (52.2%); female: 85 (47.8%) • Mean age (range): 41.58 ± 1.04 (18-72) • Grade I/II: 77 (43.3%); grade III/IV: 101 (56.7%)	• ≥4 vs. <4 • Mean NLR: 4.66 ± 0.25 • NLR ≥4: 86 (48.3%); NLR <4: 92 (51.7%)	• 17 vs. 28 months, HR 1.385, 95% CI 1.020-1.881, p=0.037 • Multivariate: no correlation	• Retrospective • No data on the extent of resection, steroid use, *MGMT* promoter methylation, and *IDH1* mutation status (patients diagnosed between 2009 and 2012) • Heterogeneity in patient population and treatment • No data on confounding variables in multivariate analysis
Kaya et al. (2017) (120)	• Total: 90 • Male: 51 (57%); female: 39 (43%) • Median age (range): 58.5 (16-93) • Grade IV: 90 (100%)	• ≥5 vs. <5 • NLR ≥5: 32 (35.6%); NLR <5: 58 (64.4%)	• 11.8 ± 4.7 vs. 15.7 ± 2.5 months, p<0.05 • Multivariate: HR 2.41, 95% CI 1.26-4.58, p<0.05	• Retrospective • Relatively small sample size • No data on steroid use, *MGMT* promoter methylation status, *IDH* mutation status, and post-surgery therapy (patients diagnosed between 2011 and 2015) • No data on confounding variables in multivariate analysis
Lopes et al. (2017) (56)	• Total: 140 (117 analyzed) • Male: 98 (70%); female: 42 (30%) • Mean age: 62.9 ± 10.0 • Grade IV: 100%	• >7 vs. ≤7 • Mean NLR: 9.48 ± 6.37	• HR 1.65, 95% CI 1.07-2.53, p=0.023 • Multivariate: HR 1.00, 95% CI 0.97-1.03, p=0.868	• Retrospective • No data on steroid use, *MGMT* promoter methylation status, and *IDH* mutation status (patients diagnosed between 2005 and 2013) • ≈50% of patients had comorbidities with potential impact on NLR • No correlation of absolute neutrophil and lymphocyte counts with overall survival • Multivariate analysis adjusted for KPS, tumor location, first-line and second-line therapy, and presence of comorbidities
Mason et al. (2017) (57)	• Total: 369 • Male: 238 (64.5%); female: 131 (35.5%) • Median age (range): 55 (18-70) • Grade IV: 100%	• ≥7.5 vs. <7.5 • Median NLR (range): 7.3 (2.8-25.3)	• HR 0.628, p<0.0001 • Multivariate: HR 1.00, 95% CI 0.70-1.44, p=0.9127	• Retrospective • Blood sampling time: postoperative • No data on *MGMT* promoter methylation and steroid use • 4.3% and 75% of patients had mutant and unknown *IDH* status, respectively (patients diagnosed between 2005 and 2013) • Multivariate analysis adjusted for age, ECOG performance status, and total TMZ cycles • Lymphocyte counts were not associated with overall survival in multivariate analysis
Wang et al. (2017) (121)	• Total: 166 • Male: 96 (58%); female: 70 (42%) • Mean age (range): 52.1 (18-80) • Grade IV: 166 (100%)	• >4 vs. ≤4 • NLR >4: 27 (16.3%); NLR ≤4: 139 (83.7%)	• 12.80 ± 2.4 vs. 6.03 ± 4.6 months, p=0.172 • Multivariate: HR 1.714, 95% CI 1.026-2.858, p=0.039	• Retrospective • No data on *MGMT* promoter methylation status and steroid use • 31 patients had mutant *IDH* (patients diagnosed between 2009 and 2014) • A disproportionate number of patients with NLR ≤4 • Multivariate analysis adjusted for age, sex, KPS, pathology, extent of resection, standard treatment, and *IDH* mutation
Wiencke et al. (2017) (122)	• Total: 72 • Male: 52 (72%); female: 20 (28%) • Median age (range): 47 (44-54) • Grade II/III: 39 (54%); grade IV: 33 (46%)	• ≥4 vs. <4 • mdNLR ≥4: 28 (39%); mdNLR <4: 44 (61%)	• 22 vs. 52 months, HR 1.78, 95% CI 1.03-3.07, p=0.038 • Multivariate: HR 2.02, 95% CI 1.11-3.69, p=0.022	• Retrospective • Relatively small sample size • No data on *MGMT* promoter methylation status • 58% of patients had *TERT* promoter mutation and 42% had *IDH* mutation • mdNLR assessed by an immunomethylomic approach was associated with survival independent of chemotherapy and steroid use • Multivariate analysis adjusted for age, grade, and mutation status
Bao et al. (2018) (123)	• Total: 219 • Male: 124 (56.6%); female: 95 (43.4%) • Aged ≥50 years: 66.7% • Grade I/II: 57 (26%); grade III/IV: 162 (74%)	• ≥2.5 vs. <2.5 • NLR ≥2.5: 162 (74%); NLR <2.5: 57 (26%)	• 12.0 ± 2.32 vs. 32 ± 5.17 months, HR 2.342, 95% CI 1.550-3.540, p<0.001 • Multivariate: HR 1.758, 95% CI 1.157-2.671, p=0.008	• Retrospective • No data on performance status, steroid use, *MGMT* promoter methylation, *IDH* mutation status, and post-surgery therapy (patients diagnosed between 2012 and 2017) • Multivariate analysis adjusted for age, sex, grade, and other markers of inflammation
Coleman et al. (2018) (124)	• Total: 100 • Male: 69 (69%); female: 31 (31%) • Median age (range): 48 (18-70) • Grade IV: 76%; grade III/IV: 24%	• ≥4 vs. <4	• HR 1.82, 95% CI 1.15-2.88, p=0.010 • Multivariate: HR 1.73, 95% CI 1.02-2.94, p=0.043	• Retrospective • Relatively small sample size • No data on *MGMT* promoter methylation and *IDH* mutation status (patients diagnosed between 2004 and 2016) • 63% were on steroids; steroid use did not modify the association between NLR and overall survival • 42 patients were on phase I trials • Multivariate analysis adjusted for steroid and antiepileptic drug use, ECOG performance status, RMH score, and trials
Wang et al. (2018) (90)	• Total: 112 • Male: 70 (63%); female: 42 (37%) • Mean age: 50 ± 12 • Grade I/II: 59 (53%); grade III/IV: 53 (47%)	• ≥4 vs. <4 • Mean NLR: 3.80 ± 1.48 • NLR ≥4: 48 (43%); NLR <4: 64 (57%)	• 20.75 ± 7.68 vs. 26.91 ± 7.50 months, HR 2.577, 95% CI 1.626-4.086, p<0.001 • Multivariate: HR 1.932, 95% CI 1.011-3.694, p=0.046	• Retrospective • No data on steroid use, *MGMT* promoter methylation, *IDH* mutation status, and post-surgery therapy (patients diagnosed between 2010 and 2013) • Multivariate analysis adjusted for tumor size, grade, KPS, and platelet-to-lymphocyte ratio
Weng et al. (2018) (92)	• Total: 105 • Male: 53 (50.5%); female: 52 (49.5%) • Mean age: 61.05 ± 12.86 and 57.74 ± 12.40 for NLR ≥4.0 and NLR <4.0 groups, respectively • Grade IV: 100%	• ≥4 vs. <4 • NLR ≥4: 44 (41.9%); NLR <4: 61 (58.1%)	• 11.23 ± 6.28 vs. 18.56 ± 11.28, p<0.001 • Multivariate: HR 1.953, 95% CI 1.255-3.039, p=0.003	• Retrospective • No data on *MGMT* promoter methylation status and steroid use • 24 patients had mutant *IDH1* (patients diagnosed between 2011 and 2014) • Multivariate analysis adjusted for age, KPS, extent of resection, full Stupp protocol, and *IDH* mutation status
Yersal et al. (2018) (114)	• Total: 80 • Male: 39 (48.7%); female: 41 (51.3%) • Mean age: 56.8 ± 13.1 • Grade IV: 100%	• >4 vs. <4 • Mean NLR: 6.3 ± 5.5	• 11.6 vs. 14.5 months, p>0.05; HR 1.258, 95% CI 0.727-2.179 p=0.412	• Retrospective • No data on performance status, *MGMT* promoter methylation status, and *IDH* mutation status (patients diagnosed between 2012 and 2017) • The post-progression salvage treatments were heterogeneous
Gan et al. (2019) (52)	• Total: 135 • Male: 89 (65.9%); female: 46 (34.1%) • Mean age (range): 70.61 ± 4.60 (65-91) • Grade III: 22 (16.3%); grade IV: 113 (83.7%)	• ≥3 vs. <3 • Mean NLR: 3.98 ± 3.28 • NLR ≥3: 65 (48.1%); NLR <3: 70 (51.9%)	• 9.6 vs. 17.1 months, HR 2.298, 95% CI 1.552-3.403, p<0.001 • Multivariate: HR 1.712, 95% CI 1.071-2.734, p=0.025	• Retrospective • No data on steroid use, *MGMT* promoter methylation status, and *IDH* mutation status (patients diagnosed between 2014 and 2018) • 51 (37.8%) did not receive any postoperative treatment • Lymphocyte counts but not neutrophil counts in isolation were prognostic • Multivariate analysis adjusted for age, sex, extent of resection, KPS, tumor grade, and therapy
Hao et al. (2019) (125)	• Total: 187 • Male: 116 (62%); female: 71 (38%) • Mean age: 55 ± 13.55 • Grade IV: 100%	• ≥4.1 vs. <4.1 • NLR (range): 4.59 ± 5.06	• HR 2.574, 95% CI 1.849-3.581, p<0.001	• Retrospective • No data on steroid use, *MGMT* promoter methylation status, and *IDH* mutation status (patients diagnosed between 2012 and 2017) • No multivariate analysis
Lv et al. (2019) (126)	• Total: 192 • Male: 113 (58.9%); female: 79 (41.1%) • Mean age: 53.25 ± 13.9 • Grade IV: 100%	• >2.7 vs. ≤2.7 • NLR >2.7: 85 (44.3%); NLR ≤2.7: 107 (55.7%)	• HR 1.650, 95% CI 1.182-2.304, p=0.003 • Multivariate: HR 0.637, 95% CI 0.454-0.894, p=0.009	• Retrospective • 37 (19.3%) patients had methylated *MGMT* promoter status; 127 (66.1%) with unknown status • No data on steroid use • 38 (19.8%) patients had mutant *IDH1*; 124 (64.6%) with unknown status (patients diagnosed between 2006 and 2018) • Multivariate analysis adjusted for age and adjuvant therapy
Maas et al. (2019) (127)	• Total: 497 (479 analyzed) • Male: 297 (59.8%); female: 200 (40.2%) • Median age (range): 62.2 (21-88) • Grade IV: 100%	• >4 vs. <4 • Median NLR (range): 6.8 (0.1-46.9) • NLR >4: 336 (67.6%); NLR <4: 143 (28.8%)	• 12.5 vs. 15.1 months, HR 1.27, 95% CI 1.01-1.58, p=0.037 • Multivariate: HR 1.11, 95% CI 0.75-1.65, p=0.607	• Retrospective • No data on *MGMT* promoter methylation status and steroid use • 20 (4%) had mutant *IDH1*; 201 (40.4%) with unknown status (patients diagnosed between 2005 and 2013) • Multivariate analysis adjusted for age, KPS, extent of resection, and therapy
Yang et al. (2019) (91)	• Total: 128 • Male: 71 (55.5%); female: 57 (44.5%) • Mean age: 47.84 ± 13.958 • Grade I/II: 67 (52.3%); grade III-IV: 61 (47.7%)	• ≥2.8 vs. <2.8 • NLR ≥2.8: 56 (43.75%); NLR <2.8: 72 (56.25%)	• 22.78 ± 3.61 vs. 48.31 ± 4.01 months; HR 2.525, 95% CI 1.611-3.957, p<0.001 • Multivariate: HR 2.037, 95% CI 1.264-3.281, p=0.003	• Retrospective • No data on steroid use, *MGMT* promoter methylation, *IDH1* mutation status, and post-surgery therapy (patients diagnosed between 2008 and 2012) • Multivariate analysis adjusted for age, grade, extent of resection, albumin, platelets, platelet-to-lymphocyte ratio, and nutritional index
Zhang et al. (2019) (128)	• Total: 188 (170 analyzed) • Male: 107 (56.9%); female: 81 (43.1%) • Age: >62 (138 (73.4%)); ≤62 (50 (26.6%)) • Grade IV: 100%	• >7.25 vs. ≤7.25 • NLR >7.25: 20 (11.8%) NLR ≤7.25: 150 (88.2%)	• Multivariate: HR 2.228, 95% CI 1.329-3.733, p=0.002	• Retrospective • No data on *MGMT* promoter methylation status and steroid use • 8 (4.4%) patients had *IDH* mutation only, 107 (59.1%) had *TERT* mutation only, and 66 (36.5%) were triple-negative (without 1p/19q codeletion, *IDH*, and *TERT* mutations); 103 (18.0%) were triple-positive (patients diagnosed between 2011 and 2016) • A disproportionate number of patients with NLR ≤7.25 • Multivariate analysis adjusted for age, extent of resection, and therapy
	• Total: 404 (358 analyzed) • Male: 228 (56.4%); female: 176 (43.6%) • Age: ≤40 (146 (36.1%)); >40 (258 (63.9%)) • Grade II-III: 100%	• >2 vs. ≤2 • NLR >2: 148 (41.3%) NLR ≤2: 210 (58.7%)	• Multivariate: HR 1.502, 95% CI 1.007-2.240, p=0.046	• Retrospective • No data on *MGMT* promoter methylation status and steroid use • 103 (26.3%) were triple-positive (1p/19q codeletion, *IDH* and *TERT* mutations), 19 (4.8%) had both *IDH* and *TERT* mutations, 100 (25.5%) had *IDH* mutation only, 48 (12.24%) had *TERT* mutation only, 78 (19.9%) were triple-negative, and 44 (11.2%) had other combinations (patients diagnosed between 2011 and 2016) • Multivariate analysis adjusted for age, grade, KPS, extent of resection, radiotherapy
Marini et al. (2020) (51)	• Total: 124 • Male: 65 (52.4%); female: 59 (47.6%) • Age: <60 (42 (33.8%)); ≥60 (82 (66.2%)) • Grade IV: 100%	• >4 vs. ≤4 • Mean NLR: 6.09 • NLR >4: 87 (70.1%); NLR ≤4: 37 (29.9%)	• HR 3.15, 95% CI 0.73-11.62, p=0.027 • Multivariate: p=0.044	• Retrospective • No data on *MGMT* promoter methylation status and steroid use • 59 (47.6%) had mutant *IDH1* (patients diagnosed between 2013 and 2019) • Multivariate analysis adjusted for age, KPS, extent of resection, adjuvant therapy, *IDH1* mutation, and hematological parameters (albumin, platelets, lymphocytes, platelet-to-lymphocyte ratio)
Garrett et al., 2021 (115)	• Total: 79 • Male: 54 (62%); female: 33 (38%) • Median age (range): 63 (51-73) • Grade IV: 100%	• >5.07 vs. ≤5.07 • NLR >5.07: 44 (55.7%); NLR ≤5.07: 35 (44.3%)	• 299 vs. 353 days, p=0.994	• Retrospective • Relatively small sample size • 23 (57.5% out of 40) had methylated *MGMT* promoter, and 5 (6.3%) had mutant *IDH* (patients diagnosed between 2013 and 2019) • 75.9% of patients received steroids, 59 (67.8%) were on pre-operative steroids at data collection
Yang et al., 2022 (129)	• Total: 208 • Male: 124 (%); female: 84 (%) • Median age (range): 58.5 (51-65) • Grade IV: 100%	• >2.1 vs. ≤2.1 • NLR >2.1: 139 (%) NLR ≤2.1: 69 (%)	• HR 2.820, 95% CI 1.992–3.993, p<0.001 • Multivariate: HR 1.769, 95% CI 1.106–2.829, p=0.017	• Retrospective • 89 (42.8%) had methylated *MGMT* promoter, • The status of *IDH1* in a cohort was wild type (patients diagnosed between 2016 and 2021) • No data on steroid use • Multivariate analysis adjusted for age, tumor location, extent of resection, KPS, radiochemotherapy, *MGMT* promoter methylation, and different blood cell counts and ratios
Hsu et al. (2022) (54)	• Total: 182 • Male: 112 (61.5%); female: 70 (38.5%) • Median age (range): 57 (18.8-79.5) • Grade IV: 69.2%; grade II/III: 30.8%	• >4 vs. ≤4 • NLR >4: 122 (67%); NLR ≤4: 60 (33%)	• Multivariate: HR 1.847, 95% CI 1.218-2.803, p=0.0039	• Retrospective • No data on performance status; 162 (89.0%) patients had unmethylated *MGMT* promoter*;* 43 (23.6%) had mutant *IDH;* 50 (27.5%) were on steroids (patients diagnosed between 2010 and 2021) • No data on confounding variables in multivariate analysis
Duan et al. (2023) (130)	• Total: 281 • Male: 155 (55.2%); female: 126 (44.8%) • Age: >65 (58 (20.6%)); <65 (223 (79.4%)) • Grade IV: 100%	• ≥2.12 vs. <2.12	• HR 1.456, 95% CI 1.286-1.649, p<0.001	• Retrospective • No data on *MGMT* promoter methylation status and steroid use • 59 (21%) patients had mutant *IDH* (patients diagnosed between 2015 and 2018) • No multivariate analysis

IDH1, isocitrate dehydrogenase 1; MGMT, O^6^-methylguanine-DNA-methyltransferase; NLR, neutrophil-to-lymphocyte ratio; TERT, telomerase reverse transcriptase.

Systematic reviews and meta-analyses have provided evidence that high baseline neutrophil count or high NLR were independently associated with adverse overall survival in various types of solid tumors (84, 117, 118, 145–151), while normalization of post-treatment NLR was associated with improved survival (84). Moreover, systematic reviews and/or meta-analyses examining the correlation between NLR and outcomes in patients treated with immune checkpoint inhibitors have reported that higher NLR is a prognostic factor of worse disease control rate, objective response rate, progression-free survival, and/or overall survival in patients with head and neck squamous cell carcinoma (152, 153), gastric carcinoma (154, 155), melanoma (156), metastatic renal cell carcinoma (157, 158), non-small cell lung carcinoma (159), hepatocellular carcinoma (160) and cancer patients in general (161–163). High post-treatment NLR has also been associated with poor survival outcomes in cancer patients treated with immune checkpoint inhibitors (162). Combining NLR assessment with other biomarkers of response to immune checkpoint inhibitors, such as PD-L1 expression, tumor mutation burden, or lymphocyte infiltration, has been shown to provide additional predictive power in identifying patients who respond to treatment (164–169). Finally, in meta-analyses, lower baseline NLR was significantly associated with immune-related adverse events (irAEs) resulting from the use of immune checkpoint inhibitors in cancer patients (170, 171).

There is no standardized cutoff for the prognostic/predictive NLR value. An NLR value ≥4 was associated with poorer overall survival in the majority of the studies of patients with glioblastoma/gliomas with statistically meaningful sample sizes (**Table 1**). Of note, in a meta-analysis of 75 eligible studies covering more than 20 cancer types, the median cutoff for high NLR with the strongest prognostic effect was 4.0 (range 1.9–7.2) (117), whereas in a prospective study in an unselected general population (individuals aged 45 years, n=8711), the reference NLR value (mean and 95% intervals) was 1.76 (0.83-3.92) (172).

Neutrophils are plastic populations of immune cells with different functions (173). In rodent models, the immunosuppressive neutrophil populations may promote tumor progression by potentiating tumor invasion, angiogenesis, and metastasis (173). In patients with cancer, the proportion of immunosuppressive neutrophils is dramatically increased (173–175). Neutrophils can suppress the activation and proliferation of cytotoxic T cells (173–176). The predominance of immunosuppressive neutrophils over lymphocytes, which demonstrate the disproportion of the CD4+/CD8+/Treg ratio, provides a clue as to why NLR is a prognostic marker of worse survival across many solid tumor types.

## 4 A composition and prognostic significance of the immune infiltrate of glioblastoma

It is worth noting that adult glioblastoma/gliomas in general differ significantly from pediatric gliomas. An overview of the composition of the tumor immune infiltrate across different types of pediatric glioma is given elsewhere (177).

### 4.1 Neutrophils

Glioblastoma tissue is abundantly infiltrated by neutrophils (178). Fossati et al. found a strong correlation between glioma tumor grade, the extent of neutrophil infiltration, and the preoperative circulating neutrophil counts (101). Over 70% of all glioma samples analyzed (*n*=105) showed significant neutrophil infiltration (40–50% of low grade gliomas and 87% of glioblastomas) (101). In another study, neutrophil infiltration was observed in 86% of II-IV glioma samples (n=232), and the level of neutrophil infiltration was significantly correlated with glioma grade (179). Increased neutrophil infiltration was associated with shorter overall survival in patients with glioblastoma (n=152) (58). Tumor-infiltrating neutrophils are an independent prognostic factor for overall survival across different tumor types (146, 180).

### 4.2 Microglia, macrophages, MDSCs

Tumor-associated macrophages and microglia are the dominant population of immune cells in the glioblastoma microenvironment, and their heterogeneity and plasticity are discussed extensively elsewhere (181). Microglia/macrophages comprise of ≈10–50% of the glioblastoma mass (182–186). Both M1- and M2-like microglia/macrophages (differentiated by pro-inflammatory and anti-inflammatory polarization/phenotype states, respectively) have been detected in human gliomas (72, 187–194). However, it should be noted that the M1/M2 dichotomy is oversimplified. MDSCs also infiltrate glioblastoma (73, 74, 195, 196). Detailed flow cytometry analysis revealed that MDSCs, microglia, and macrophages constituted approximately 40%, 40%, and 20% of the glioblastoma mass, respectively, and that glioblastoma-associated myeloid cells presented a continuum between the M1- and M2-like phenotypes, with closer alignment to the non-polarized M0 macrophage phenotype (72).

Ionized calcium-binding adaptor molecule-1 (IBA-1) is a pan-marker for all microglia and macrophages. High IBA-1 intensity was correlated with longer survival (193). However, in another study, the number of IBA+ cells was positively correlated with the overall tumor size and edema but not with overall survival (72). CD204+ (scavenger receptor) (193) or CD163+ (scavenger receptor) (194) M2-like microglia/macrophage density was correlated with worse survival, whereas lower expression of CD163 and higher expression of CCL3 (C-C Motif Chemokine Ligand 3), an M1 marker, was correlated with better survival (192). In contrast, Zeiner et al. found that high levels of CD68+ (a pan-macrophage marker), CD206+ (mannose receptor C type 1), and CD163+ tumor-infiltrating macrophage subpopulations in the vital tumor core of patients with *IDH1*R132H-non-mutant glioblastoma (n=241) were associated with improved survival (187). Finally, Karimi et al. revealed that increased levels of MPO+CD163−P2Y12−CD68+ macrophages were associated with prolonged survival of patients with glioblastoma (184).

### 4.3 NK and NKT cells

NK cells and invariant NKT cells are scarcely present in glioblastoma/glioma tissue (60, 183, 184), and the role of these immune cell subtypes has not been clearly established in patients with glioblastoma/glioma. Nevertheless, there is increasing evidence of NK or NKT cell-based immunotherapy efficacy in rodent glioma models (197, 198). In addition, a local administration of activated haploidentical NK cells (199) or irradiated CAR-NK cells (NK-92/5.28. z) targeting HER2 (200) in patients with recurrent glioblastoma was feasible and safe.

### 4.4 Dendritic cells

There are plasmacytoid dendritic cells, type 1 and type 2 classical dendritic cells, monocyte-derived dendritic cells, and a new dendritic cell subset, DC3 (201). The heterogeneity and functionality of the dendritic cell compartment in patients with glioblastoma (subsets, counts, and functionality) are poorly characterized and reviewed elsewhere (202).

### 4.5 T lymphocytes

The density of tumor-infiltrating CD4+, CD8+, and Tregs increases with glioma grade (203–206), with higher levels of infiltrated CD4+ cells than CD8+ cells (203, 207). However, Innocenti et al. reported no difference between CD4+ and CD8+ cell counts in glioblastoma tissue samples (n=59) (208). In general, tumor-infiltrating lymphocytes are differentially distributed in glioblastoma samples, from absent to abundant (183, 209–211). T cell infiltrates are mainly located in the perivascular areas and zones of tumor invasion into the surrounding brain parenchyma and are only infrequently found within the tumor tissue and in the perinecrotic areas (211–215). The percentages of immunological synapses established by tumor-infiltrating lymphocytes with tumor cells are very low (213), and the tumor-infiltrating lymphocytes have a suppressed and functionally impaired state/phenotype (described as tolerance and exhaustion) (36, 37, 60, 69, 216, 217). These observations indicate that although the density of infiltrated lymphocytes varies considerably between patients, tumor-infiltrating lymphocytes cannot readily migrate into the immunosuppressive tumor microenvironment, are mainly arrested in the perivascular or peritumoral space, and are functionally compromised.

There is no clear association between tumor-infiltrating lymphocytes (CD3+, CD4+, or CD8+ T cell infiltrates) and overall survival in patients with glioblastoma/glioma (**Table 2**) (58, 60, 203, 205, 208–210, 218–230). In addition, based on the FOXP3+ phenotype alone, there was no correlation between overall survival and Treg infiltration in univariate and multivariate analyses of most studies (203–205, 225, 226, 231–233). In meta-analyses, inconsistent correlations between different tumor-infiltrating lymphocyte subsets and overall survival have also been reported for other types of solid cancer (234–243). Cytotoxic CD8+ T cells and memory CD45RO+ T cells are strongly correlated with good outcomes in most cancer types, whereas the prognostic value of Th2, Th17, Tregs, MDSCs, macrophages, and NK populations is inconsistent and varies depending on the cancer type, stage, or study (234–243).

**Table 2:** Correlation between the density of tumor-infiltrating lymphocytes and overall survival of patients with glioblastoma/gliomas.

Study (year)	Patient characteristics	T cell subtype	Correlation with overall survival	Limitations/comments
Brooks et al. (1978) (218)	• Total: 149 • Samples were collected from 1962 through 1976	• Lymphocytes	• Positive	• Retrospective • Samples were collected from 50^th^ to 70^th^ years • Lymphocytes were identified morphologically • Heterogeneity in patient cohorts and treatments
Palma et al. (1978) (219)	• Total: 200 • Male: 128; female: 72 • Samples were collected from 1952 through 1973	• Lymphocytes • Graded as definite: 23 (11.5%); slight: 46 (23%); absent: 131 (65.5%)	• Positive	
Schiffer et al. (1979) (220)	• Total: 324 • Mean age: 50.6 ± 10.7 • Grade IV: 269; grade III: 55	• Lymphocytes	• No correlation	
Böker et al. (1984) (221)	• Total: 199	• Lymphocytes	• Positive	
Safdari et al. (1985) (222)	• Total: 342	• Lymphocytes	• Negative	
Rossi et al. (1989) (223)	• Total: 68	• CD4+ or CD8+	• No correlation	
Yang et al. (2010) (210)	• Total: 108 • Grade IV: 100%	• CD8+	• Positive • No multivariate analysis	• Retrospective • No data on other prognostic variables
Lohr et al. (2011) (205)	• Total: 44 • Male: 29 (65.9%); female: 15 (34.1%) • Mean age: 58.1 ± 11.29 • Grade IV: 100%	• CD8+	• Positive • No multivariate analysis	• Retrospective • Relatively small sample size • No data on other prognostic variables
Kim et al. (2012) (224)	• Total: 61 • Male: 32 (52.5%); female 29 (47.5%) • Median age (range): 59 (14-80) • Grade IV: 100%	• CD3+, CD4+ or CD8	• No correlation	• Retrospective • Relatively small sample size
Kmiecik et al. (2013) (60)	• Total: 65 • Grade IV: 100%	• CD3+ or CD8+	• Positive • No multivariate analysis	• Retrospective • Relatively small sample size
Rutledge et al. (2013) (209)	• Total: 171 (from The Cancer Genome Atlas, TCGA) • Grade IV: 100%	• CD3+ • Graded as absent: 93 (54%); present: 59 (35%); abundant: 19 (11%)	• No correlation	• Retrospective
Yue et al. (2014) (225)	• Total: 62 • Male: 43 (69.4%); female: 19 (30.6%) • Median age (range): 56 (13-77)	• CD8+	• No correlation; HR 1.15, 95% CI 0.69-1.93, p=0.597	• Retrospective • Relatively small sample size
Han et al. (2014) (203)	• Total: 90 • Male: 46 (51.1%); female: 44 (48.9%) • Mean age: 45.7 ± 13 • Grade IV: 100%	• CD4+ or CD8+	• No correlation	• Retrospective • Relatively small sample size • The number of CD4+ and CD8+ cells did not vary significantly according to age, sex, preoperative KPS, degree of resection, tumor size, and *MGMT* promoter methylation
		• High CD4+/low CD8+	• mOS 255 vs 568 days, p<0.001 • Multivariate: HR 1.508, 95% CI 1.162-1.956, p=0.002	• Multivariate analysis adjusted for age, KPS, and *MGMT* promoter methylation status
Han et al. (2015) (58)	• Total: 152 • Male: 95 (62.5%); female: 57 (37.5%) • Mean age: 50.4 ± 15.4 • Grade IV: 152 (100%)	• CD3+	• No correlation	• Retrospective
Madkouri et al. (2017) (226)	• Total: 186 • Male: 105 (56.5%); female: 81 (43.5%) • Median age (range): 64 (29-89) • Grade IV: 100%	• CD8+	• HR 0.47, 95% CI 0.32-0.7, p=0.0001 • Multivariate: HR 0.59, 95% CI 0.39-0.91, p=0.01	• Retrospective • IL-17A+ T cells were associated with a poorer OS • Foxp3 cells were associated with a good prognosis • Multivariate analysis adjusted for sex, age, KPS, and surgery
Orrego et al. (2018) (227)	• Total: 43 • Male: 22 (51.2%); female 21 (48.8%) • Median age (range): 47 (8-74) • Grade IV: 100%	• CD3+ or CD8+ • Graded as mild (71.8%), moderate (25.6%), marked (2.6%)	• No correlation	• Retrospective • Relatively small sample size • Necrosis was ubiquitously present in samples • Lymphocyte intensity, distribution, and presence in perivascular area were not associated with preoperative KPS, *MGMT* promoter methylation or degree of resection
		• CD4+	• Negative; univariate and multivariate analysis (p<0.05)	
Wang et al. (2021) (228)	• Total: 57 • Male: 45 (78.9%); female: 12 (21.1%) • Mean age: 55.3 ± 8.9 • Grade IV: 100% (multifocal and multicentric)	• Low CD8+	• 12.5 vs. 6.3 months; HR 3.671, 95% CI 1.679-8.026, P=0.001 • Multivariate: HR 4.404, 95% CI 1.954-9.926, P=0.0004	• Retrospective • Relatively small sample size • Multivariate analysis adjusted for KPS, age, *MGMT* promoter methylation status, extent of resection, tumor size, and radio-/chemotherapy
Mauldin et al. (2021) (229)	• Total: 77 • Male: 45 (58.4%); female: 32 (41.6%) • Mean age: 61.48 ± 14.68 • Grade IV: 100%	• CD4+ or CD8+	• No correlation	• Retrospective • Relatively small sample size • No significant associations between dexamethasone treatment and CD4+ or CD8+ densities • Multivariate analysis adjusted for KPS, extent of resection, and *MGMT* promoter methylation status
		• CD8/CD4 ratio	• Multivariate: HR 0.31, 95% CI 0.14-0.71, p=0.005	
		• CD8+Ki67+	• HR 0.36, 95% CI 0.2-0.66, p=0.001 • Multivariate: HR 0.15, 95% CI 0.06-0.38, p<0.001	
		• CD8+T-bet+	• HR 0.46, 95% CI 0.26-0.79, p=0.004, • No multivariate analysis	
Innocenti et al. (2023) (208)	• Total: 59 • Male: 34 (58%); female: 25 (42%) • Median age (range): 62 (26-80) • Mean age: 62.15 ± 10.9 • Grade IV: 100%	• CD4+	• HR 1.79, 95% CI 1.1-3.1, p*=*0.035 • No multivariate analysis	• Retrospective • Relatively small sample size • Multivariate analysis adjusted for age, gender, *MGMT* promoter methylation status, extent of resection, and radio-/chemotherapy
		• CD8+ or CD4/CD8 ratio	• No correlation	
		• Low CD4+ and low CD8+	• Multivariate: HR 0.38, 95% CI 0.18-0.79, p=0.014	
Sobhani et al. (2023) (230)	• Total: 58 • Male: 36 (62%); female: 22 (38%) • Median age (range): 66 (41–81) • Grade IV: 100%	• CD3+ • Graded as absent: 10 (17%); mild: 37 (64%); moderate/high: 11 (19%)	• No correlation	• Retrospective • Relatively small sample size

KPS, Karnofsky performance score; MGMT, O^6^-methylguanine-DNA-methyltransferase; OS, overall survival.

### 4.6 CD4+ and CD8+ T lymphocytes are highly heterogeneous immune cell populations

The inconsistent correlations between tumor-infiltrating lymphocytes and survival (**Table 2**) may stem from substantial functional and phenotypic complexity and plasticity of CD4+ and CD8+ T cell subsets discovered by high-dimensional single-cell mass cytometry (cytometry by time-of-flight, CyTOF) (244). Detailed qualitative and quantitative assessments of tumor immune infiltrates using mass cytometry to measure the frequencies and ratios of immune cell subsets and their functional and activation status may be required for accurate prognostication (184, 245–247). Distinct subsets of T cells are anti-inflammatory, pro-inflammatory, or both, and a dual role in cancer immunity has been ascribed to CD3+CD4+ cell subsets (246). For example, in follicular B-cell lymphomas, 12 subsets of intratumoral CD4+ T cells were identified, and specific subpopulations were correlated with poor or improved patient survival (246). In patients with clear cell renal cell carcinoma, 22 subsets were identified among infiltrating T cell lymphocytes, and a distinct immune composition correlated with survival (247). In addition, different prognoses may be obtained depending on the degree of infiltration of immunosuppressive FOXP3(high) Tregs or non-suppressive FOXP3(low) cells (248). For example, colorectal carcinomas with abundantly infiltrated FOXP3(low) T cells demonstrated a much better prognosis than tumors with predominant FOXP3(high) Treg infiltration (248).

It is also evident that not only the density of tumor-infiltrating lymphocytes but also the ratio between immunosuppressive immune cell subsets and cytotoxic lymphocytes have an effect on prognostic significance. In some studies, increased CD8+/CD4+, CD4+/CD8+, CD3+/Treg, or CD8+/Treg ratios, rather than the absolute counts of individual populations, were correlated with survival outcomes in patients with glioblastoma/glioma (203, 229, 232). Furthermore, the immunosuppressive cellular immune landscape in the glioblastoma/glioma microenvironment goes beyond well-defined immune cell subtypes such as Tregs, neutrophils, MDSCs, and tumor-associated macrophages (215, 249). Li et al. revealed that immunosuppressive CD3+CD4+FOXP3− type 1 regulatory T cells occurred at high frequencies within glioblastoma tissue (249). In addition, Waziri et al. found that a great portion of CD3+ T cells within glioblastoma tissue was represented by CD3+CD4+CD56+ T cells (215). However, these T cells did not represent classical invariant NKT cells as they were neither stained with antibodies against an invariant TCR Vβ24 nor with CD1d tetramer loaded with α-Gal-Cer (215). These CD3+CD4+CD56+ T cells might be immunosuppressive, since immunosuppressive CD3+CD4+CD56+CD25+FOXP3+ T cells were identified at a high frequency in hepatocellular carcinoma, and higher infiltration of these cells was inversely correlated with survival (250).

### 4.7 The need to standardize the assessment of tumor-infiltrating lymphocytes

A lack of standardization and recommendations for assessing tumor-infiltrating lymphocytes (technical and methodological differences associated with staining and analysis, including high inter‐observer variability) in glioma tissue, as well as non-uniform patient cohorts, are potential factors contributing to inconsistent correlations between tumor infiltrating lymphocytes and overall survival (**Table 2**). It is known that pre-analytical variables such as tissue collection, fixation (time, buffer composition, and temperature), processing (dehydration reagents, temperature, and paraffin embedding), storage, staining (manual *versus* automated, quality, and quantity of antibodies), and other conditions may affect the accurate assessment of CD3+, CD4+, and CD8+ lymphocytes within a tissue (251, 252). Moreover, since the immune infiltrate and individual subpopulations of immune cells are heterogeneously distributed in glioblastoma tissue (e.g., central areas *versus* marginal areas; perivascular areas *versus* perinecrotic areas), histochemical sections of different tumor regions will give different insights into immune cell density and diversity. New high‐throughput multiplex immunohistochemistry and immunofluorescence technologies are expanding the ability to obtain additional information on cellular composition and spatial arrangement with greater reproducibility using standardized quantitative protocols (253). Therefore, in-depth spatial immunophenotyping of glioblastoma tissue, together with consensus guidelines for the assessment of glioma-infiltrating lymphocytes, is required to more accurately establish the prognostic power of infiltrating lymphocytes. In general, this also applies to subsets of myeloid cells (254, 255).

### 4.8 The immune landscape of isocitrate dehydrogenase-mutant gliomas

Gliomas with *IDH* mutation exhibit a unique immune landscape due to the role of the oncometabolite *R*-2-hydroxyglutarate (2-HG) in glioma immune evasion (256–258). The infiltration of CD3+ (259), CD4+ (260), CD8+ (260, 261) T lymphocytes, Tregs (262), monocyte-derived macrophages (263), neutrophils (260), overall CD45+ immune cells, including macrophages, dendritic cells, and T cells (264) was reduced in *IDH*-mutant gliomas compared with *IDH*-wild type gliomas. However, another study documented a higher relative abundance of dendritic cells and CD8+ T lymphocytes in *IDH*-mutant gliomas compared with *IDH*-wild type gliomas (265). The activity of tumor-infiltrating immune subsets may be significantly compromised by *R*-2-hydroxyglutarate, which was shown to impair monocyte differentiation into dendritic cells and dendritic cell functionality (265), proliferative potential and effector functions of T cells (266–269), and NK cell-mediated killing (270), promote an immunosuppressive phenotype of macrophages (271) and reduce intratumoral vasculature density (272). Due to a distinct immune microenvironment, including distorted immune cell infiltration and impaired immune cell activity, *IDH*-mutant gliomas may be potentially more resistant to immunotherapy. Small-molecule IDH inhibitors may reverse *R*-2-hydroxyglutarate mediated immune suppression and sensitize *IDH*-mutant gliomas to immunotherapy (256, 273, 274). Moreover, in a double-blind phase III trial in patients with grade 2 *IDH*-mutant glioma (NCT04164901), vorasidenib, an inhibitor of mutant IDH1/2 enzymes, was shown to significantly improve progression-free survival compared with the placebo group (median progression-free survival, 27.7 *versus* 11.1 months) (275).

### 4.9 The immune landscape and prognostic significance of mesenchymal, proneural, and classical molecular subtypes

In 2006, using unsupervised expression profiling on a cohort of 76 grade III and IV astrocytoma samples to classify tumors into prognostic groups, Philips et al. defined subtypes based on a 35-gene signature termed proneural, mesenchymal, and proliferative (276). The proneural subtype, containing the majority of grade III astrocytoma samples, was distinguished by markedly better prognosis compared with other subtypes (276). In 2010, Verhaak et al. described a gene expression-based molecular classification (840-gene set classifier) of glioblastoma samples into the proneural, neural, classical, and mesenchymal subtypes (77% subtype concordance) (277). Somatic mutations and/or DNA copy number aberrations/overexpression of *EGFR*, *NF1*, and *PDGFRA/IDH1* predominantly defined the classical, mesenchymal, and proneural subtypes, respectively. *MGMT* promoter methylation status was not associated with subtypes (277). In meta-analyses, *IDH* mutations are associated with better progression-free and overall survival in patients with glioma (278–280). Surprisingly, overall survival of patients with the proneural subtype was not significantly different from other gene expression subtypes defined according to the classification of Verhaak et al. (281, 282). However, Noushmehr et al. found the glioma cytosine-phosphate-guanine (CpG) island methylator phenotype (G-CIMP) (281). Tumors with this phenotype were predominantly of the proneural subtype and were strongly associated with *IDH1* mutations, and proneural G-CIMP-positive patients had significantly better survival than proneural G-CIMP-negative patients or all other non-proneural patients in univariate and multivariate analysis (281). Later, Turcan et al. (283) and Brennan et al. (284) confirmed that the survival advantage of the proneural subtype was conferred by G-CIMP status, strongly associated with *IDH*-mutant gliomas, with proneural non-G-CIMP and other subtypes demonstrating similar and less favorable outcomes. Further studies showed that *IDH*-mutant G-CIMP-positive tumors with 1p/19q codel status were associated with better overall survival than *IDH*-mutant G-CIMP-positive tumors with 1p/19q non-codel status (285, 286), and G-CIMP-low non-codel subgroup, based on the extent of DNA methylation, had poorer outcome compared with G-CIMP-high non-codel subgroup in *IDH*-mutant gliomas (287–290). Finally, by combining epigenetic signature and gene copy number variations, Li et al. separated *IDH*-mutant glioblastoma into G-CIMP-high group and G-CIMP-low group without *CDKN2A* and *MET* alteration with favorable and comparable overall survival, while G-CIMP-low group with *CDKN2A* and/or *MET* alteration showed significantly shorter overall survival in univariate and multivariate analysis (290). Currently, the combination of these parameters allows for improved prediction of outcome (291).

In 2017, applying unsupervised transcriptome analysis after filtering glioblastoma overexpressed genes to 369 *IDH*-wild type glioblastoma samples, Wang et al. confirmed three subtypes previously designated mesenchymal, proneural, and classical (≥93% subtype concordance; defined 50-gene signatures for each subtype with overlap with Verhaak et al.’s 840-gene set ranging from 42% to 54%), while the neural phenotype was suggested to be non-tumor specific (292). In addition, strong associations between subtypes and genomic abnormalities in previously reported subtype-defining genes were also confirmed (292). Again, patient survival did not differ significantly between the three subtypes (292). In 2019, using a 500-gene set classifier (only 108 genes matched Verhaak et al.’s 840-gene set) on six different datasets (three TCGA-cohorts and three Asian-cohorts), Teo et al. also confirmed the classical, mesenchymal, and proneural subtypes, with similar survival outcomes between subtypes (293). Finally, in 2023, White et al. also found no significant difference in overall survival between subtypes in the GLIOTRAIN (n=123), TCGA (n=164), and CGGA (n=693) cohorts after stratifying the mesenchymal, proneural, and classical subtypes according to Wang et al.’ report (294). Interestingly, the authors introduced novel glioblastoma tumor microenvironment subtypes for *IDH* wild type glioblastoma (TME^High^, TME^Med^, and TME^Low^), characterized by significantly different expression of genes specific to all immune and endothelial cell markers. However, stratification into these new subtypes showed no association with overall survival in the GLIOTRAIN, TCGA, CGGA, and DUKE cohorts (294).

Using The Cancer Genome Atlas (TCGA) database, Doucette et al. analyzed the mRNA expression levels of immune system genes (cytokines, cell markers, and signaling pathways) between four glioblastoma subtypes and found that the mesenchymal subtype was the most proinflammatory due to the preferential enrichment of both pro-inflammatory and immunosuppressive genes compared with other subtypes (295). Rutledge et al., using histochemical analysis, found that tumor-infiltrating lymphocytes were enriched in the mesenchymal subtype and strongly associated with mutations in *NF1* and *RB1*, while lymphocytes were depleted in the classical subtype, *EGFR*-amplified, and homozygous *PTEN*-deleted tumors. However, no association with survival was found (209). Wang et al. documented the increased presence of macrophages/microglia and neuroglial cells in mesenchymal subtype, and NF1 deactivation was associated with macrophage/microglia recruitment (292). The activated natural killer cell gene signature was significantly reduced in the mesenchymal subtype, the resting memory CD4+ T cell gene signature was significantly reduced in the proneural subtype, and the activated dendritic cell gene signature was significantly greater in the classical subtype (292). In an immunohistochemical study of the immune infiltrate of the four glioblastoma subtypes, Martinez-Lage et al. found that the mesenchymal and proneural subtypes were the most and least immunogenic, respectively (194). The percentage of CD3+, CD4+, and CD5+ lymphocytes differed significantly between the mesenchymal and proneural or classical subtypes but not other subtypes. The percentage of CD8+ did not differ between the four subtypes. The percentage of CD163+ and CD68+ macrophages/microglia in the mesenchymal subtype was also significantly different compared with the classical or proneural subtypes, with no difference between them (194). In another immunohistological study of three glioblastoma subtypes defined according to the classification of Wang et al., the numbers of IBA1+ microglia/macrophage cells and CD3+ and FOXP3+ lymphocytes were significantly higher in the mesenchymal subtype compared with other subtypes, with no significant difference between the proneural and classical subtypes (296). The number of CD8+ differed significantly only between the mesenchymal and proneural subtypes (296). On the contrary, Han et al. reported that the number of CD4+, CD8+, and FoxP3+ lymphocytes did not vary greatly between the subtypes defined according to the classification of Verhaak et al. (203). Finally, using flow cytometry, Gabrusiewicz et al. found that the frequency of MDSCs, microglia, and macrophages in the proneural and neural subtypes was not significantly different; the classical subtype had a markedly higher percentage of MDSCs than macrophages, whereas the mesenchymal subtype was predominantly infiltrated with microglia (72).

Taken together, glioma/glioblastoma transcriptome subtypes in different studies were defined using different numbers of only partially overlapping gene sets, and in each case the subtypes (except for the classification of Philips et al.) did not differ prognostically unless at least proneural G-CIMP status was not taken into account, despite the fact that the proneural subtype is characterized by the presence of prognostic *IDH* mutations much more often than other subtypes, on the one hand, and the existence of the greatest immunological difference between the proneural/*IDH* mutant and mesenchymal subtypes, on the other hand. These observations might explain why stratification of glioblastoma patients based on the transcriptomic subtypes has not translated into clinical practice. Considering the enormous inter- and intratumoral cellular heterogeneity and plasticity, as well as the evolution of the cancer genome and phenotype, clinically meaningful subtyping of glioblastoma based on the transcriptome alone is challenging. However, there is some retrospective clinical evidence that mesenchymal or TME^High^ glioblastoma might respond better to immunotherapy (vaccine, checkpoint inhibitor or oncolytic virus) (294, 297).

## 5 Future directions

**Figure 1:**
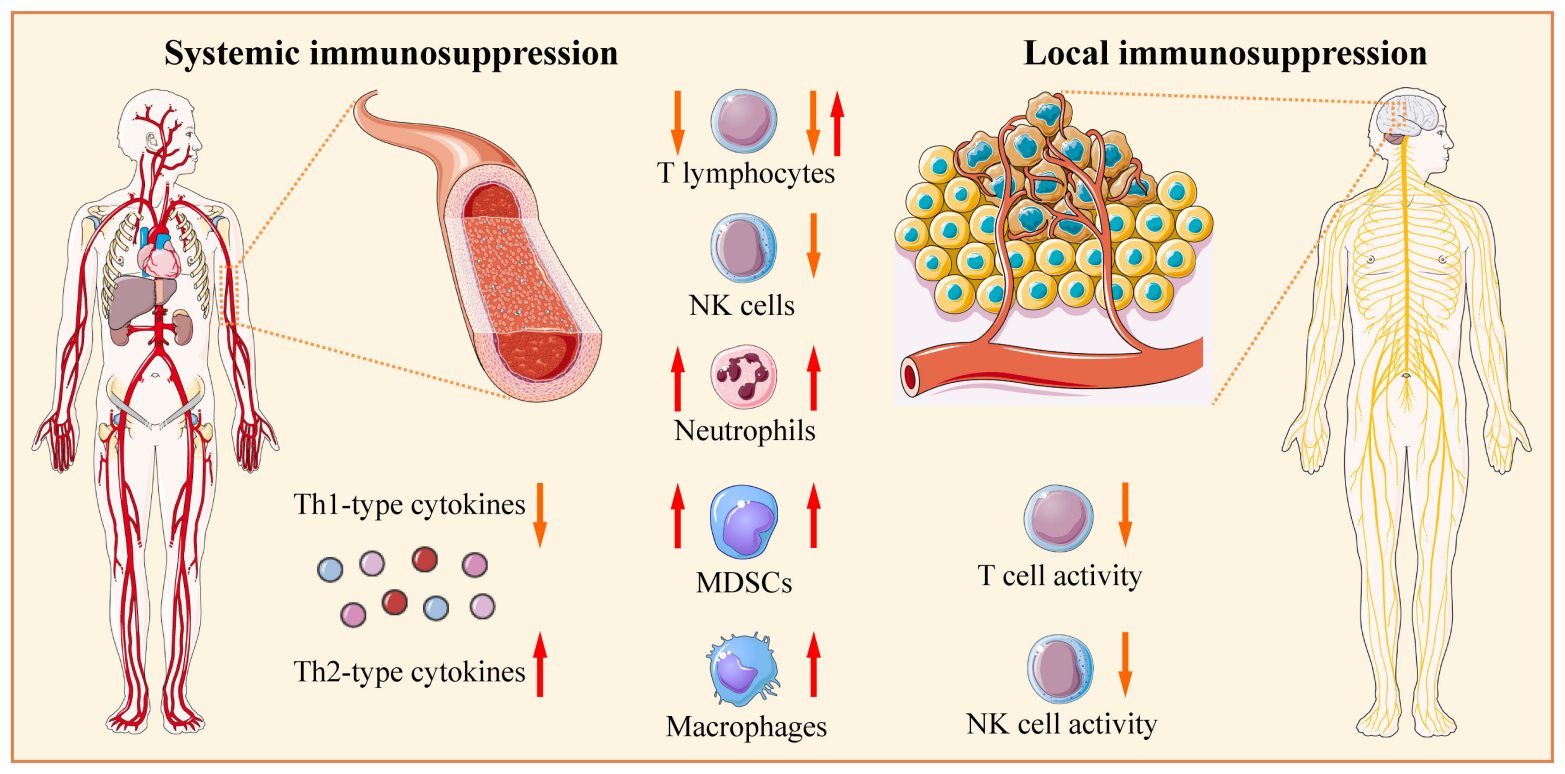
Glioblastoma/glioma-related systemic and local immunosuppression. Before standard therapy, patients may have reduced counts of circulating T lymphocytes (lymphopenia); however, in the majority of studies, the increased fractions of regulatory T cells (Tregs) are documented. The numbers of circulating myeloid-derived suppressor cells (MDSCs) and neutrophils are higher in patients with glioblastoma than in healthy donors or patients with low-grade gliomas. The counts of circulating natural killer (NK) and NKT cells are within the normal range in the majority of studies. The density of tumor-infiltrating CD4+, CD8+, and Tregs increases with glioma grade; however, lymphocytes are differentially distributed in tissue samples, from absent to abundant. Glioblastoma tissue is abundantly infiltrated by neutrophils and macrophages, while NK cells and NKT cells are scarcely present. Serum levels of Th1-type cytokines (IL-2, IL-6, IL-12, TNF-α, and IFN-γ) are reduced, while serum levels of Th2-type cytokines (IL-4 and IL-10) are increased. Serum, cerebral spinal fluid, or tumor cyst fluid from patients can suppress the proliferation and/or function of lymphocytes and other immune cells from healthy donors. Consequently, patients have defective functions of antigen-presenting, helper, and effector immune cell subsets due to altered expression of different soluble and membrane proteins (receptors, costimulatory molecules, and cytokines). The tumor-infiltrating lymphocytes have suppressed and functionally impaired state/phenotype. The Figure was generated using Servier Medical Art, provided by Servier, licensed under a Creative Commons Attribution 3.0 unported license.

There is growing interest in evaluating neoadjuvant immunotherapy in neuro-oncology (298–300). Neoadjuvant immunotherapy is advantageous over adjuvant immunotherapy, since it is applied before lymphotoxic standard radio-/chemotherapy (43). However, in patients with glioblastoma/glioma before standard therapy, tumor-related immunosuppression involves deregulation of many components of immunity, including changes in expression of different soluble and membrane proteins, reduced T lymphocyte counts (lymphopenia), increased NLR, increased levels or ratios of circulating and tumor-infiltrating immunosuppressive immune subsets (e.g., macrophages, MDSCs, and Tregs), and defective functions of antigen-presenting, helper, and effector immune cell subsets due to altered expression of receptors, costimulatory molecules, and cytokines (**Figure 1**). In this condition, the effectiveness of neoadjuvant immunotherapy for glioblastoma/glioma might be suboptimal.

The spatial complexity and phenotypic diversity of the immune infiltrate (multicellular spatial organization) in patients with glioblastoma/gliomas at presentation, as well as the prognostic and/or predictive significance of immune cell subsets/signatures remain poorly characterized and largely unknown (255, 301, 302). As discussed above, the accurate prognostic assessment of immune infiltrate by traditional immunochistochemistry is influenced by too many analytical variables and factors. Although multidimensional single-cell approaches such as multiplexed ion beam imaging, imaging mass cytometry, and mass cytometry has become powerful tools for characterizing different immune cell subsets in immunology and identifying specific immune signatures associated with survival and response to immunotherapy in cancer patients (184, 245–247, 303–306) and may also help better decipher immune deregulation in patients with glioblastoma/gliomas and identify putative predictive or prognostic markers of therapeutic response and improved survival, but at present they should still be considered discovery tools in preclinical research and clinical trials rather than for routine clinical practice (303–305, 307–312). It is also worth considering the factor of the availability and quality of tissue for assessing spatially distributed immune infiltrate in glioblastoma. Moreover, in various reports, approximately 15-20% of cases present as unresectable glioblastoma (diagnostic “biopsy-only”) (313). However, the actual number of such patients may be higher. In the National Cancer Database, the percentage of patients with unresectable glioblastoma among patients diagnosed from 2004 to 2013 with known survival and extent of resection is 28.5% (314). Kole et al. documented that among 1325 patients with biopsy-only glioblastoma who received radiochemotherapy, the median overall survival was 9.2 months (313), and Harlay et al. reported that among 139 patients who underwent biopsy only, 54 (39%) and 68 (49%) were amenable to standard radiochemotherapy or chemotherapy only, with the median overall survival of 14 months (95% CI, 9.65-18.71) and 8 months (95% CI, 4.62-7.67), respectively (315). This relatively large group of patients is particularly in need of new treatment strategies and should not be ignored.

What is needed is a cost-effective, easily accessible and repeatedly validated prognostic and predictive factor, available to all patients, that can be dynamically assessed (at baseline, during and/or after treatment) and rapidly integrated into all ongoing and planned clinical studies of various forms of therapy in all clinical centers around the world. In general, immune-related biomarkers derived from blood rather than tumor tissue may be more suitable in terms of these requirements. NLR has prognostic value in patients with solid tumors across cancer types, stages of disease, and treatment strategies, including immunotherapy. However, although NLR has often been correlated with the effectiveness of immune checkpoint inhibitors in carcinomas, NLR has only rarely been taken into account when evaluating the effectiveness of vaccines, oncolytic viruses, and other immunotherapies in cancer patients. For patients with glioblastoma, this may also be due to the fact that the vast majority of clinical trials of all forms of therapy in general are non-randomized and/or uncontrolled (316, 317). It is also important to note that evaluation of the prognostic/predictive significance of NLR has been largely limited to retrospective studies, with very few of the studies based on prospectively collected samples [e.g (318–320)], including glioblastoma/glioma (53, 112). Since the number of prospective studies evaluating NLR in oncology is limited, NLR has not yet entered routine clinical practice as a stratification/predictive factor.

The prognostic/predictive power of NLR may be further improved by combining NLR assessment with other biomarkers. According to recent meta-analyses and systematic reviews, absolute/total lymphocyte count (ALC/TLC, especially post-treatment) (321), platelet-to-lymphocyte ratio (PLR) (133, 322), lymphocyte-to-monocyte ratio (LMR) (323), systemic immune-inflammation index (SII), calculated by platelet count×neutrophil count/lymphocyte count (129, 324), and systemic immune response index (SIRI), calculated by neutrophil count×monocyte/lymphocyte count (325–327) are the emerging prognostic factors in glioblastoma/glioma. In a retrospective study, Yang et al. developed and compared the SII-NLR, SII-PLR, and NLR-PLR, and SII-NLR-PLR scoring systems and found that the combination of these inflammatory markers demonstrated greater predictive accuracy for overall survival at one and two years than any single indicator in patients with glioblastoma (n=208), with the best scoring system being SII-NLR (129). The authors constructed a nomogram including age, Karnofsky Performance Status (KPS), extent of resection, *MGMT* promoter methylation status, chemoradiotherapy, and SII-NLR score to predict 2-year survival in patients with glioblastoma (the c-index of the nomogram was 0.848 (95% CI 0.836–0.861) and 0.843 (95% CI 0.830–0.855) excluding *MGMT* promoter methylation status) (129).

Finding methods to reduce NLR is another important area of research in clinical oncology. In patients with recurrent glioblastoma (n=18), treatment with recombinant interleukin-7 restored and maintained total lymphocyte counts without serious toxicity and irrespective of steroid and temozolomide use (328). Interleukin-7 is currently considered the most potent therapeutic candidate for the treatment of lymphopenia in cancer and non-cancer patients (41). As we have discussed, NLR may be affected by many physiological and pathological confounding factors, including psychological/emotional stress in cancer patients. It is known that cortisol and epinephrine may increase neutrophil and decrease lymphocyte counts (329–331). In a randomized trial of lung cancer patients (n=80), psychological intervention was shown to significantly reduce NLR compared with usual care (332).

## 6 Conclusion

Glioma progression and molecular characteristics (e.g., *IDH* mutations or mesenchymal gene signature) have distinct effects on major immune cell subsets, and conversely, different proportions of immune cell subsets and their polarization or activation states may have different effects on tumor progression, response to therapy, and survival. All attempts to identify a reliable prognostic immune marker in glioblastoma tissue have led to markedly contradictory results, which can be explained, among other things, by the unprecedented level of spatial heterogeneity of the immune infiltrate and the significant phenotypic and functional diversity of immune subpopulations. High NLR has been repeatedly established as an independent prognostic factor for shorter overall survival in patients with glioblastoma and carcinomas, and its combination with other markers of the immune response significantly improves the accuracy of prediction; however, more prospective studies are needed to confirm the prognostic/predictive power of NLR. We call for incorporating the dynamic assessment of NLR and other emerging blood inflammatory markers (e.g., platelet-to-lymphocyte ratio, lymphocyte-to-monocyte ratio, systemic immune-inflammation index, and systemic immune response index) into all neuro-oncology trials to carefully evaluate and compare their individual and combinatorial prognostic/predictive significance and relative superiority.

## Author contributions

AAS: Conceptualization, Funding acquisition, Investigation, Writing – original draft, Writing – review & editing. AOS: Writing – review & editing. MPV: Writing – review & editing. AAC: Writing – review & editing. OVA: Writing – review & editing. KAP: Writing – review & editing. VPC: Writing – review & editing, Supervision.
